# The Value of Colonoscopy in Assessing Rectal Bleeding in Patients Referred From Outpatient Care Units in Erbil, Iraq

**DOI:** 10.7759/cureus.71911

**Published:** 2024-10-20

**Authors:** Rawand Haweizy, Farman N Qader

**Affiliations:** 1 Surgery, College of Medicine, Hawler Medical University, Erbil, IRQ; 2 General Surgery, Kurdistan Board of Medical Specialties, Erbil, IRQ

**Keywords:** colonoscopy, colorectal cancer, hemorrhoids, inflammatory bowel disease, rectal bleeding

## Abstract

Background and objectives: Rectal bleeding denotes bleeding from the lower colon or rectum, specifically from a location distal to the ligament of Treitz. Lower gastrointestinal bleeding (LGIB) is very common in adults of all ages. This study aimed to review the diagnostic findings of colonoscopy in outpatients who had nonurgent rectal bleeding and identify common pathologies causing rectal bleeding in patients at Erbil and Rizgary Teaching Hospitals in Iraq.

Methods: We selected 400 male and female patients from outpatient departments with evidence of rectal bleeding, referring them to the gastroenterology units of Erbil and Rizgary Hospitals in Erbil, Iraq. Patients with upper gastrointestinal bleeding (UGIB) sources and other symptoms were excluded from the study. We prepared the bowel, subjected all patients to a colonoscopy, and recorded the findings. Where necessary, biopsies were also taken. A diagnosis was made based on the colonoscopic findings.

Results: The total number of patients was 400. Their mean age + SD was 44.9 + 15.9 years, ranging from 18 to 83 years, and the highest proportion of the diagnoses was hemorrhoids, either alone (n = 140, 35%) or in combination with other diseases. The results indicated that 48 (40%) males aged < 45 years had hemorrhoids, compared with 33 (33.7%) males aged ≥ 45 years; for females, 29 (30.9%) in those aged < 45, compared to 30 (34.1%) in those aged ≥ 45. The next two prevalent diagnoses in the total sample were inflammatory bowel disease (n = 31, 7.8%) and colorectal tumor (n = 37, 9.3%). It was observed that around one-fifth (n = 79, 19.8%) of the sample was normal.

Conclusion: This study highlights the leading causes of LGIB as diagnosed through colonoscopy. The main causes and patterns of LGIB across gender and age groups in Erbil were generally similar to other settings but with some differences. Hemorrhoids caused the most rectal bleeding, followed by tumors and then inflammatory bowel disease. The older group had more cases of diverticulosis than the younger group. Although most findings were benign, colonoscopy should be strongly considered for outpatients (young and old) with nonurgent rectal bleeding.

## Introduction

The term lower gastrointestinal bleeding (LGIB) was originally used to refer to bleeding that comes from a source away from the ligament of Treitz [1,2]. When patients complain about hematochezia (passage of maroon or bright red blood or blood clots per rectum), they are suspected to have LGIB. About 85% of LGIB cases involve the colon, 10% of upper gastrointestinal bleeding (UGIB) cases result in hemorrhagia, and 3% to 5% of this bleeding affects the small intestines [3]. According to most people, LGIB is intermittent and often self-limiting [4]. Benign anal conditions, such as hemorrhoids or anal fissures, have been reported in most patients with rectal bleeding; however, rectal bleeding can also be associated with inflammatory bowel disease (IBD) or colorectal cancer as their common symptom [4,5].

The potential causes of rectal bleeding can be identified by the patient’s age, such that IBD, anal fissures, and hemorrhoids are more likely to afflict younger patients under 30 years, while a higher risk of colorectal cancer has been reported in patients over the age of 50 years suffering from rectal bleeding. About 20% to 30% of all patients with major gastrointestinal (GI) bleeding are likely to develop LGIB [6,7]. Lower gastrointestinal bleeding has been reported to cause limited, enormous, and life-threatening amounts of blood loss; however, self-limited bleeding and uncomplicated hospitalization have been reported in most patients [7,8].

Colonoscopy aims to diagnose and, if necessary, treat the bleeding source in hemodynamically stable patients [9,10]. According to numerous studies, colonoscopy can reveal definitive bleeding sites in more than 70% of patients [5,7]. In recent years, the use of colonoscopy has increased. Assessing the entire colon through colonoscopy provides the opportunity to acquire tissue biopsy and facilitate therapeutic intervention [11]. A colonoscopy needs to be performed for a complete evaluation of the large bowel in cases where the bleeding source cannot be confirmed or if anemia and bleeding continue [12]. Most doctors might recommend conducting a limited evaluation of the anorectosigmoid area for patients who are younger than 40 years old because only less than 5% of colorectal cancer cases have been reported in this age group [13]. Moreover, anorectal diseases, such as hemorrhoids or anal fissures, have been referred to as the main leading cause of rectal bleeding in patients below the age of 30 years [14]. Suspecting a lower GI source of bleeding necessitates evaluating all cases, with proctosigmoidoscopy and colonoscopy serving as the most accurate imaging methods for the lower GI tract [15,16].

Causes of rectal bleeding have been divided into different groups: neoplastic, inflammatory (infectious, idiopathic, and radiation-induced), vascular (angiodysplasia, hemorrhoids, and ischemic), and anatomic (diverticulosis). There is limited research from the Erbil governorate and Kurdistan Region of Iraq about the main causes of rectal bleeding and the role of colonoscopy in evaluating rectal bleeding cases. Therefore, this study aimed to review the diagnostic findings of colonoscopy in outpatients who had nonurgent rectal bleeding and identify common pathologies causing rectal bleeding in these patients.

## Materials and methods

Study design and setting

Data were collected retrospectively from the endoscopy unit database, highlighting all consecutive flexible colonoscopy examinations over five years (2014 to 2018). This research is a cross-sectional descriptive study comprising 400 patients and was conducted at the Department of Gastroenterology at the Erbil and Rizgary Teaching Hospitals in Iraq. These two hospitals are the main public hospitals in Erbil city and contain the main gastroenterology departments. Gastroenterology services are also provided in a number of smaller public hospitals within the Erbil governorate and in most private hospitals. The study protocols were approved by the Research Ethics Committee of the College of Medicine, Hawler Medical University (approval no. 12/4). Institutional permission was obtained from Rizgary Teaching Hospital’s administration department (approval no. 238).

The inclusion criteria were male and female patients aged 12 to 80 years who presented with visible bleeding per rectum as their main complaint. The exclusion criteria were patients below the age of 12 or above 80 years, those with a possible UGIB site, those with bleeding per rectum because of acute infectious bloody diarrhea, and those with altered bowel habits, constipation, weight loss, and melena. The study included patients who presented in outpatient departments or were admitted to the Erbil and Rizgary Hospitals. All data were collected from hardware document files of patients from the gastroenterology units of the two hospitals. Records with missing data were excluded from the analysis. The study was conducted with the hospitals’ acceptance to provide the data, under their supervision, and according to the hospital guidelines.

All the patients were prepared for colonoscopy by colon emptying preparations using Colon Clean (ethyl glycol) a day before the examination. They were administered enema once the night before and again on the examination day. Midazolam 3 mg and pethidine 25 mg were used as the anxiolytic and analgesic, respectively. Olympus colonoscopes (Olympus Corp., Hachioji, Tokyo, JPN) were used for the examinations. The colonoscopy results were analyzed using statistical tests. Lesions with some degree of suspicion were biopsied and sent to a laboratory for histopathological studies. This study did not include the analysis of the histopathological results, as they were not included in the patient's records. The frequencies and percentages of independent variables of age and gender were calculated, and their association with the dependent variable of colonoscopy findings was analyzed.

Statistical analysis

Data were analyzed using SPSS Statistics version 22 (IBM Corp., Armonk, NY, USA). Categorical variables were presented as frequencies and percentages, and numerical variables were summarized by means and standard deviations. We used Fisher’s exact test to compare proportions, as over 20% of cells had a value of less than 5. A p-value ≤ 0.05 was considered statistically significant.

## Results

The total number of patients in the study sample was 400, ranging from 18 to 80 years (mean age + SD: 44.9 + 15.9 years). Table 1 shows that the highest proportion of patients (n = 99, 24.8%) were in the age group 30 to 39 years, and the lowest proportion were those less than 18 years old. More than half of the sample (n = 218, 54.5%) were males.

**Table 1:** Age and gender distribution of the studied sample

Variable	No.of participants (total n = 400)	Percentage
Age (years)		
< 30	73	18.3
30 to 39	99	24.8
40 to 49	78	19.5
50 to 59	60	15.0
≥ 60	90	22.5
Gender		
Male	218	54.5
Female	182	45.5

Table 2 indicates that around one-fifth (n = 79, 19.8%) of the sample were normal: 38 (17.4%) males and 41 (22.5%) females. Hemorrhoids comprised the most significant proportion of the diagnoses, occurring either alone in 140 (35%) patients or in conjunction with other diseases. The next few prevalent diagnoses were tumors (n = 37, 9.3%), IBD (n = 31, 7.8%), polyp (n = 22, 5.5%), and nonspecific colitis (n = 22, 5.5%). The other diagnoses were rare, as presented in Table 2.

**Table 2:** Final diagnosis by gender IBD: Inflammatory bowl disease

Diagnosis	Males (n = 218)		Females (n = 182)		Total (n = 400)	
	No.	Percentage	No.	Percentage	No.	Percentage
Hemorrhoids	81	37.2	59	32.4	140	35.0
Normal	38	17.4	41	22.5	79	19.8
Tumor	17	7.8	20	11.0	37	9.3
IBD	19	8.7	12	6.6	31	7.8
Polyp	16	7.3	6	3.3	22	5.5
Nonspecific colitis	15	6.9	7	3.8	22	5.5
Hemorrhoids + polyp	12	5.5	3	1.6	15	3.8
Diverticulitis	5	2.3	9	4.9	14	3.5
Solitary rectal ulcer	5	2.3	9	4.9	14	3.5
Fissure	1	0.5	8	4.4	9	2.3
Hemorrhoids + diverticulitis	2	0.9	3	1.6	5	1.3
Hemorrhoids + fissure	2	0.9	1	0.5	3	0.8
Fissure + nonspecific colitis	2	0.9	0	0.0	2	0.5
Hemorrhoids and nonspecific colitis	1	0.5	1	0.5	2	0.5
Polyp + diverticulitis	1	0.5	1	0.5	2	0.5
Fissure and polyp	0	0.0	1	0.5	1	0.3
Polyp + nonspecific colitis	0	0.0	1	0.5	1	0.3
Hemorrhoids + solitary rectal ulcer	1	0.5	0	0.0	1	0.3
Total	218	100.0	182	100.0	400	100.0

Table 3 shows that 48 (40%) males aged < 45 years had hemorrhoids, compared with 22 (33.7%) males aged ≥ 45 years, but it is evident that nine (9.2%) older males had polyps in addition to the hemorrhoids. Only 13 (13.3%) older males were normal, compared with 25 (20.8%) younger males. The prevalence of IBD was 12.5% (n = 15) among younger males, compared to 4.1% (n = 4) among older males. Tumors were present in 15 (15.3%) older males, compared with two (1.7%) younger males. The table also indicates that five (5.1%) older males had diverticulitis as opposed to the younger males.

**Table 3:** Final diagnosis by age among males Note: The p-value is determined by using Fisher’s exact test. IBD: Inflammatory bowel disease

Diagnosis	< 45 years (n = 120)		≥ 45 years (n = 98)		Total (n = 218)		p-value
	No.	Percentage	No.	Percentage	No.	Percentage	
Hemorrhoids	48	40.0	33	33.7	81	37.2	0.001
Normal	25	20.8	13	13.3	38	17.4	
IBD	15	12.5	4	4.1	19	8.7	
Tumor	2	1.7	15	15.3	17	7.8	
Polyp	9	7.5	7	7.1	16	7.3	
Nonspecific colitis	10	8.3	5	5.1	15	6.9	
Hemorrhoids + polyp	3	2.5	9	9.2	12	5.5	
Diverticulitis	0	0.0	5	5.1	5	2.3	
Solitary rectal ulcer	2	1.7	3	3.1	5	2.3	
Fissure + nonspecific colitis	0	0.0	2	2.0	2	0.9	
Hemorrhoids + diverticulitis	1	0.8	1	1.0	2	0.9	
Fissure	1	0.8	0	0.0	1	0.5	
Hemorrhoids + fissure	1	0.8	0	0.0	1	0.5	
Hemorrhoids & nonspecific colitis	1	0.8	0	0.0	1	0.5	
Polyp + diverticulitis	1	0.8	0	0.0	1	0.5	
Hemorrhoids + solitary rectal ulcer	1	0.8	0	0.0	1	0.5	
Total	120	100.0	98	100.0	218	100.0	

It is evident in Table 4 that 22 (23.4%) younger females (< 45 years) were normal, compared with 19 (21.6%) older women (≥ 45 years). Eight (8.5%) younger women had fissures, while none of the older women had fissures. The table also presents the other diagnoses.

**Table 4:** Final diagnosis by age among females Note: The p-value was determined by using Fisher’s exact test. IBD: Inflammatory bowel disease

Diagnosis	< 45 years (n = 94)		≥ 45 years (n = 88)		Total (n = 182)		p-value
	No.	Percentage	No.	Percentage	No.	Percentage	
Hemorrhoids	29	30.9	30	34.1	59	32.4	0.400
Normal	22	23.4	19	21.6	41	22.5	
Tumor	11	11.7	9	10.2	20	11.0	
IBD	6	6.4	6	6.8	12	6.6	
Diverticulitis	4	4.3	5	5.7	9	4.9	
Solitary rectal ulcer	4	4.3	5	5.7	9	4.9	
Fissure	8	8.5	0	0.0	8	4.4	
Nonspecific colitis	3	3.2	4	4.5	7	3.8	
Polyp	2	2.1	4	4.5	6	3.3	
Hemorrhoids + diverticulitis	1	1.1	2	2.3	3	1.6	
Hemorrhoids + polyp	1	1.1	2	2.3	3	1.6	
Hemorrhoids + fissure	1	1.1	0	0.0	1	0.5	
Fissure and polyp	1	1.1	0	0.0	1	0.5	
Hemorrhoids and nonspecific colitis	0	0.0	1	1.1	1	0.5	
Polyp + diverticulitis	0	0.0	1	1.1	1	0.5	
Polyp + nonspecific colitis	1	1.1	0	0.0	1	0.5	
Total	94	100.0	88	100.0	182	100.0	

## Discussion

We conducted the present study to determine the frequency of different causes of bleeding in patients suffering from rectal bleeding and LGIB, given the significance of colonoscopy in evaluating these patients. The patients’ mean age was 44.9 years, with most (n = 228, 57%) belonging to the age group of over 40 years. Allen et al., who studied rectal bleeding in adults over 40 years, concluded that rectal bleeding is prevalent in this age group [17]. The results of Chait’s [18] study, which indicated an increase in LGIB rate with age, also agree with the findings of the present study, which included a higher proportion of patients over 40 years. A larger number of the patients (n = 218, 54.5%) in the present study were also males. This finding aligns with that of Zahmatkeshan et al.’s [19] study on the etiology of LGIB in children, which indicated that males experienced LGIB more frequently than females (59.2% vs. 40.8%). Similar results of a higher prevalence of LGIB among males have also been reported by other studies conducted in different parts of the world [20].

Lower gastrointestinal bleeding is one of the most frequent complaints of patients in outpatient clinics. It differs from UGIB in terms of epidemiology, prognosis, and management and is typically nonurgent and self-limiting [4]. It is usually self-limiting and involves benign anal conditions, such as hemorrhoids or anal fissures [5]. According to the present study, colonoscopy had a diagnostic yield of 85.7%. Chaudhry et al.’s study, which introduced colonoscopy as the initial test for acute LGIB, also revealed an acceptable accuracy similar to the present study [15].

Studies have indicated that LGIB can be initiated due to various causes in different parts of the world. For instance, researchers have identified diverticular disease and vascular ectasia as the most common causes of LGIB in Western Europe, especially among young and middle-aged individuals [21]. However, the most common finding in the present study was hemorrhoids in 37.2% (n = 81) and 32.4% (n = 59) of the males and females, respectively. The high prevalence of hemorrhoids as a cause of LGIB in this study could be related to having a lower prevalence of diverticular disease in this setting and also the delay or stigma in seeking treatment for hemorrhoids in this culture, which end up having LGIB. Similar to the present study, a Jordanian review found 701 patients diagnosed with hemorrhoids as the most common cause of rectal bleeding [22]. Alruzug et al. [23] also found that the most common colonoscopic findings for LGIB were hemorrhoids (38.5%), followed by diverticulosis (12.1%), and malignant neoplasm (9.9%).

Regarding gender, the present study’s colonoscopy findings revealed that more males under 45 years old had hemorrhoids compared to those above 45. However, the former group had more normal findings and fewer cases of IBD, tumors, polyps, nonspecific colitis, hemorrhoids, and polyps. The colonoscopy findings indicated a significant difference between these two age groups of males (p = 0.001). The disparate frequency of hemorrhage causes observed across the aforementioned age groups could be attributed to having different causes of LGIB in different age groups, with younger patients having more benign lesions such as hemorrhoids and fissures while older patients being more prone to tumors with the advancing age.

We obtained similar results for the female patients: those ≥ 45 years of age had more tumors, IBD, diverticulitis, nonspecific colitis, polyps, and hemorrhoids, but there was no significant difference between the two age groups (p-value > 0.05). Previous research has demonstrated that older people with LGIB are more likely to have underlying causes such as hemorrhoids, polyps, and nonspecific colitis [18], which is supported by the present study’s results.

Hemorrhoids, IBD, tumors, and polyps were found to be the four most frequent causes of LGIB in male patients, whereas the four most common causes of LGIB in females were hemorrhoids, tumors, IBD, and diverticulitis. These findings are in line with a previously conducted study, which also identified hemorrhoids, IBD, and tumors as the most frequent causes of LGIB in males and females, while polyps were more common in males and diverticulitis in females. [24].

In the comparisons of patients below 45 years with those ≥ 45 for both genders, the results of the present study revealed that hemorrhoids, IBD, polyps, nonspecific colitis, and fissures were more common among the patients below 45 years, while tumors, hemorrhoids along with polyps, and fissures along with nonspecific colitis were more prevalent among patients ≥ 45 years. In the female patients, those who were younger than 45 years had more tumors and fissures, while those who were over 45 years had more hemorrhoids, diverticulitis, solitary rectal ulcer, nonspecific colitis, and polyps. Makaju et al. also revealed a similar pattern and disparate frequency of hemorrhage causes observed across the different age groups and in both males and females [25].

About 9.3% of the patients with rectal bleeding (37 females and 17 males) had colorectal tumors. Moreover, we found IBD in 8.7% (n = 19) of the males, 12.5% (n = 15) of those under 45 years old, and 4.1 (n = 4) of those over 45 years old; these figures were 6.4% (n = 6) and 6.8% (n = 6) in the females, respectively. In the present study, IBD was found to be the third cause of LGIB in males, accounting for 7.8% (n = 31) of the cases, while it was the fourth cause in females with 6.6% (n = 12). This finding aligns well with the findings of Acosta et al.’s study, which revealed that IBD accounts for 6% to 16% of cases of rectal bleeding [26]. In the present study, nonspecific colitis was responsible for 6.9% (n = 15) and 3.8% (n = 7) of the LGIB cases in males and females, respectively. However, Goenka et al. reported that ulcerative colitis is the most common cause of rectal bleeding, followed by polyps, colonic carcinoma, and colonic tuberculosis [27]. The present study is the first study from Erbil governorate in Iraq to determine the main causes of LGIB and evaluate the role of colonoscopy in the diagnosis. It determines the main hemorrhage causes in males and females and across two age groups. While the most common causes of LGIB bleeding in Erbil city were similar to other settings, there are a number of causes that are more or less common in our locality that contributed to new knowledge in this field. 

Limitations

The study was conducted in only two public hospitals in Erbil, Iraq; hence, we missed several cases from the private sector and other hospitals in the public sector. Moreover, the sample may not reflect the general population. Furthermore, not all colonoscopies were done by the same physician, which may have affected the overall assessment.

As our data collection was done retrospectively, a key limitation of this study is the overall low quality of documentation on which the analysis is based, as well as the large number of clinical variables throughout the hospital course that were not documented. Poor documentation is a persistent research challenge in our region. The patient records only included age and gender as demographic characteristics. Other demographic and patient characteristics and behaviors, such as BMI, diet, consumption of vegetables and fruit, amount of water consumed, and history of constipation, could have helped in a better analysis and could explain the high prevalence of hemorrhoids, especially among younger patients.

## Conclusions

This study highlights the leading causes of LGIB as diagnosed through colonoscopy. The main causes and patterns of LGIB across gender and age groups in Erbil were generally similar to other settings but with some differences. Hemorrhoids were the most common cause of LGIB in both men and women of all ages. Colorectal tumor, a serious problem, was the third most common cause, but they were more common in people over 45 years. Inflammatory bowel disease was more common in people younger than 45. Colonoscopy was found to be a valid and reliable diagnostic method for patients who suffer from and complain about rectal bleeding. Therefore, this method is highly recommended for all patients with rectal bleeding, particularly the elderly, who are more prone to developing serious complications.
